# HMGB1 dysregulation: a neuroimmune bridge to cognitive impairment in autoimmune thyroiditis

**DOI:** 10.3389/fimmu.2026.1764288

**Published:** 2026-02-17

**Authors:** Jue Wang, Gaoping Chu, Longfei Ding, Wenqing You, Bin Liu, Haibo Xue

**Affiliations:** 1 Department of Endocrinology and Metabolism, Binzhou Medical University Hospital, Binzhou, China; 2 Department of Dermatology, Binzhou Medical University Hospital, Binzhou, China; 3 Department of Neurology, Binzhou Medical University Hospital, Binzhou, China; 4 Medical Research Center, Binzhou Medical University Hospital, Binzhou, China

**Keywords:** astrocytes, CD4^+^ T cells, cognitive impairment, Hashimoto’s thyroiditis, HMGB1, microglial activation, neuroinflammation

## Abstract

**Background:**

Cognitive and affective disturbances are frequent extra-thyroidal manifestations of Hashimoto’s thyroiditis (HT), even in euthyroid patients, with severe cases progressing to Hashimoto’s encephalopathy. The mechanisms underlying these CNS complications are still unclear; however, neuroinflammation—driven by CD4^+^ T cells and Hmgb1-mediated glial activation—is increasingly implicated. To elucidate this link, we explore in an experimental autoimmune thyroiditis (EAT) model whether Hmgb1 amplifies immune pathways to exacerbate cognitive and emotional impairments.

**Methods:**

In C57BL/6 mice, EAT was induced by multiple injections of pTg. Histopathological analysis and ELISA confirmed the induction of thyroiditis. Exploratory behavior was assessed in an open field test, and associative memory was evaluated using the novel object recognition task, Y-maze, and Morris water maze. PCR was performed to detect inflammatory markers indicative of neuroinflammation. Furthermore, Western blotting was used to assess Hmgb1 release, and immunofluorescence (IF) was employed to examine the cytoplasmic translocation of Hmgb1 in brain sections, as well as the morphology and activation markers of microglia and astrocytes.

**Results:**

Mice with EAT, despite preserved systemic thyroid hormone levels, displayed significant deficits in both spatial and recognition memory. Histological and immunofluorescence analyses revealed pronounced activation of microglia in the cortex and hippocampus, accompanied by an increased number of A1-like astrocytes and disrupted polarization of AQP4. Infiltrating CD4^+^ T cells were detected in these regions and were found to secrete IL-17A. Neuroinflammatory changes were associated with elevated Hmgb1 expression and increased numbers of CD68^+^ microglia, as confirmed by co-localization analyses. Pharmacological inhibition of Hmgb1 markedly reduced microglial activation and alleviated cognitive impairments.

**Conclusions:**

Our results identify Hmgb1 as a key factor that translates peripheral thyroid autoimmunity into central neuroinflammation. It functions as a driving force behind pathogenic glial and Th17/IL-17A responses, which propagate neurotoxicity and lead to cognitive-affective dysfunction. Targeting Hmgb1 may thus offer a viable therapeutic approach to prevent or treat neurological symptoms associated with HT.

## 1 Introduction

Hashimoto’s thyroiditis (HT) is a common autoimmune disorder characterized by thyroidal lymphocyte infiltration and elevated anti-TPO and anti-Tg antibodies, often resulting in hypothyroidism and systemic complications. Among these, Hashimoto’s encephalopathy (HE) is a rare but potentially debilitating manifestation, presenting with cognitive deficits, psychiatric symptoms, depression, and occasionally seizures. Although its pathophysiology remains unclear, immune-mediated neuroinflammation and molecular mimicry between thyroid autoantibodies and brain antigens are thought to play central roles, potentially disrupting neural circuits involved in mood regulation and cognition (1–3). Neuroinflammation is a key driver in the pathogenesis of many central nervous system (CNS) disorders, including neurodegenerative diseases, psychiatric conditions, and acute neurological events. It is well established that systemic chronic inflammation is a major trigger of neuroinflammatory responses, which can lead to intact blood–brain barrier (BBB) disruption and subsequent infiltration of peripheral immune cells into the CNS (4–9). Neuroinflammation in the brain is driven by the synergistic action of resident microglia and infiltrating immune cells, notably CD4^+^ T cells of the Th1 and Th17 lineages (10, 11). These cells release key pro-inflammatory cytokines such as IL-17A, IFN-γ, and TNF-α. The resulting activation of microglia and astrocytes fosters a sustained neuroinflammatory environment, which underlies subsequent neuronal injury, synaptic dysfunction, and cognitive decline (12–15).

Notably, the hippocampus and prefrontal cortex—key regions for memory, learning, emotional regulation, and executive function—are particularly vulnerable to neuroinflammatory processes. Interconnected through neural circuits that facilitate memory retrieval and cognitive processing, these regions play a central role in the pathophysiology of neuropsychiatric disorders (16, 17). High mobility group box 1 (HMGB1), a prototypical damage-associated molecular pattern (DAMP), has emerged as a pivotal mediator of neuroinflammation. Released actively by immune cells (including microglia) or passively from stressed or damaged cells, HMGB1 engages pattern-recognition receptors such as TLR4 and RAGE, promoting downstream inflammatory signaling, inflammasome activation, and cytokine production. Through these pathways, HMGB1 has been implicated in inflammatory neuronal injury and neurodegenerative processes across multiple CNS disease contexts, including traumatic brain injury, hemorrhagic injury, and Alzheimer’s disease (18–21). Increased HMGB1 expression has been observed in patients with autoimmune thyroiditis (AIT), correlating with both thyroid dysfunction and systemic inflammation (22). Despite this, the role of HMGB1 in mediating the thyroid–brain axis—particularly in the setting of autoimmune thyroiditis and its associated neuropsychiatric complications—has not been fully elucidated. In the present study, we employ an experimental autoimmune thyroiditis (EAT) mouse model to investigate the mechanisms by which thyroid autoimmunity drives neuroinflammation. We further explore how HMGB1 modulates immune responses and contributes to dysfunction in both the thyroid and the brain, with the goal of identifying potential therapeutic targets to prevent or treat neurological complications associated with HT.

## 2 Materials and methods

### 2.1 Animals

Female C57BL/6 mice (6–8 weeks old; 16–20 g) were purchased from the Jinan Pengyue Experimental Animal Breeding Center. They were housed under controlled conditions at the Animal Center of Binzhou Medical University Hospital (Binzhou, China), including a 12-hour light/dark cycle, standard mouse chow, and a stable temperature and humidity environment. This study was approved by the Institutional Animal Care and Use Committee of Binzhou Medical University Hospital (20241108-14).

### 2.2 Animal model establishment

**Figure 1:**
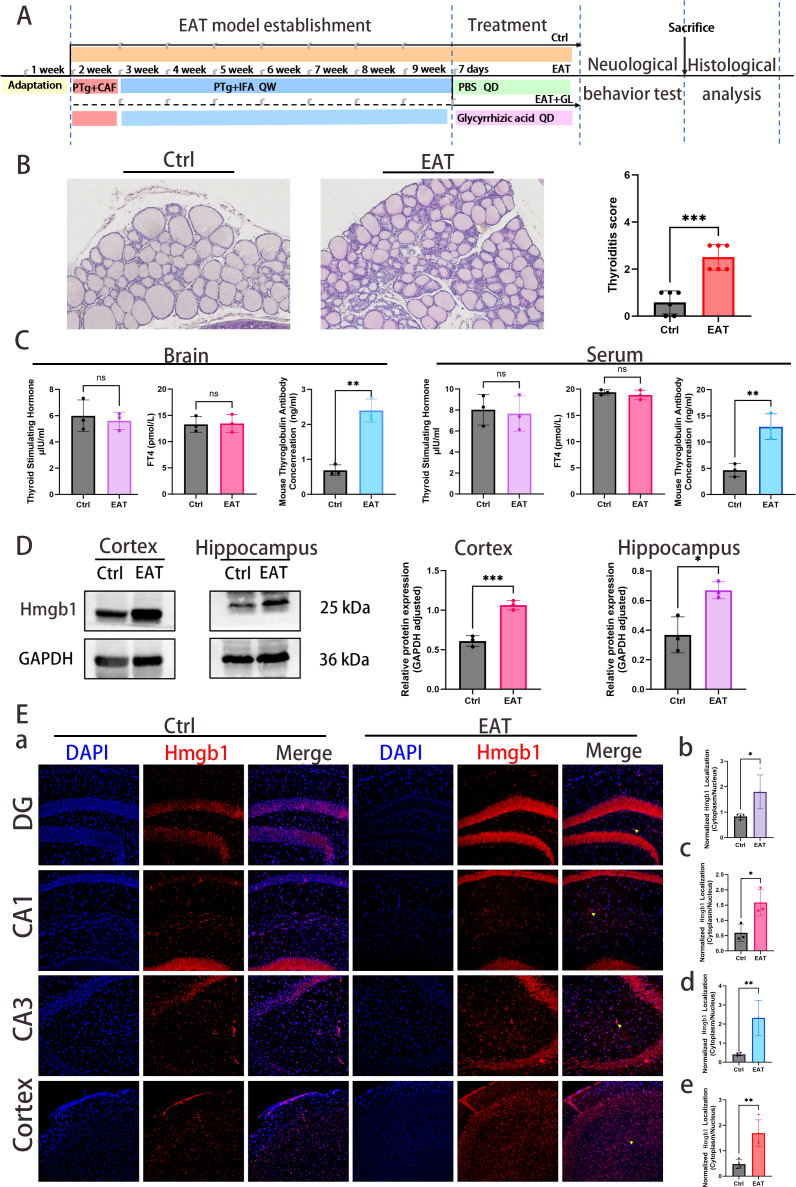
Schematic diagram of the experimental design **(A)**. Representative hematoxylin and eosin (H&E) staining (×20) of thyroid tissue **(B)**. Quantification of mononuclear cell infiltration shows significantly greater lymphocytic infiltration in the thyroids of PTg-injected mice compared with normal controls. Levels of TSH, FT4 and TgAb measured in serum and brain homogenate supernatants, respectively **(C)**. Western blot analysis showing elevated Hmgb1 expression in the hippocampus and cortex **(D)**. Quantification of Hmgb1 levels in the cortex and hippocampus is shown. IF staining shows nuclear translocation of Hmgb1 (red) in the cortex and hippocampal subregions (CA1, CA3, and DG), with nuclei counterstained by DAPI (blue) **(Ea)**. Quantification of Hmgb1 translocation between EAT and control groups in DG **(Eb)**, CA1 **(Ec)**, CA3 **(Ed)**, and cortex **(Ee)**. Data are presented as mean ± s.e.m., n = 3 per group. *P ≤ 0.05; **P ≤ 0.01; ***P ≤ 0.001 by Student’s t-test.

A total of 36 eight-week-old untreated mice were randomly divided into three groups. After a one-week acclimatization period, 12 mice were randomly selected for the control group (Ctrl), while the remaining mice were used to establish the EAT model after the same acclimatization period. EAT was induced by multi-site subcutaneous (s.c.) immunization. Mice were immunized with 100 μg of pTg (Sigma, T1126) emulsified in Freund’s adjuvant—complete (Sigma, F5881-6X) in the first week and incomplete (Sigma, F5506-6X) from the second to the eighth week—to establish the EAT model, with twelve mice randomly selected as the model group (EAT) (23). Following EAT induction, mice were randomly allocated to the EAT+GL group and received glycyrrhizin (50 mg/kg; MCE, HY-N0184), a functional inhibitor of Hmgb1, via intraperitoneal (i.p.) injection once daily for 7 consecutive days (n = 12) and administered at a fixed time window each day (24). The experimental timeline is illustrated in **Figure 1A**.

### 2.3 Open field

To assess anxiety-like behavior and exploratory activity, an open-field test was performed in a square arena (45 × 45 cm) with 40-cm-high opaque walls. Each mouse (n = 12 per group) was placed in the same corner at the beginning of the test and allowed to explore freely for 5 min while behavior was recorded from above. Videos were analyzed using the JL Behv behavioral video analysis system (Shanghai Jiliang Software Technology Co., Ltd., China). Total distance traveled and time spent in the central ROI were quantified as indices of locomotor activity and anxiety-like behavior. The central ROI was defined as a 25 × 25 cm square in the center of the arena, and all ROIs were kept identical across groups.

### 2.4 Y-maze test

Spatial working memory was assessed using a Y-maze consisting of three identical arms positioned 120° apart. Mice (n = 12 per group) were placed at the end of one arm and allowed to explore freely for 10 min while behavior was recorded from above and analyzed using Any-maze. An arm entry was counted when the mouse’s center point crossed into an arm. Spontaneous alternation was defined as consecutive entries into three different arms. The alternation rate (%) was calculated as: Alternation rate (%) = (number of correct alternations/(total arm entries − 2)) × 100.

### 2.5 Novel object recognition memory test

Recognition memory was assessed using the novel object recognition (NOR) task in the same arena (45 × 45 cm). Mice were habituated to the empty arena for 2 consecutive days. On day 3 (training), mice were allowed to explore two identical objects for 10 min. After 24 h (test), one familiar object was replaced with a novel object, and mice explored for 10 min. Object exploration time was quantified using the JL Behv system. Exploration was defined as the mouse’s nose oriented toward the object within 2 cm for at least 0.5 s; climbing or sitting on the object was not counted as exploration. The discrimination index (DI) was calculated as: DI = (Time with novel − Time with familiar)/(Time with novel + Time with familiar), reflecting preference for novelty.

### 2.6 Morris water maze task

Spatial learning and memory were assessed using the Morris water maze. The circular pool (diameter 1.5 m; height 0.5 m) was filled with water maintained at 20 ± 1 °C and rendered opaque with white nontoxic dye. A hidden platform (10 cm diameter) was submerged 1 cm below the water surface in one quadrant. Mice were trained for 5 consecutive days to locate the platform, and swim paths were tracked automatically using Any-maze. Escape latency, path length, and swim speed were recorded during training. On day 6, a probe trial was performed with the platform removed, and the number of crossings over the former platform location and/or time spent in the target quadrant within 60 s were recorded to evaluate memory retention.

### 2.7 Anesthesia, perfusion, and tissue processing

Mice were deeply anesthetized with isoflurane (induction 3% in oxygen; maintenance 1.5% via a nose cone) delivered using an isoflurane vaporizer. Oxygen flow was set to 0.5–1.0 L/min. Depth of anesthesia was confirmed by loss of righting reflex and absence of pedal withdrawal reflex prior to perfusion, and animals were kept warm on a heating pad throughout the procedure. Mice were then transcardially perfused via the left ventricle: for experiments requiring fresh tissues, perfusion was performed with ice-cold sterile PBS to remove intravascular blood and tissues were collected immediately for immune cell isolation, protein extraction, and RNA analysis; for experiments requiring fixed tissues, mice were perfused with PBS followed by 4% paraformaldehyde for *in situ* fixation, and tissues were subsequently processed for histology and immunostaining. Death was confirmed by cessation of respiration and heartbeat after perfusion.

### 2.8 Hematoxylin–eosin staining

After behavioral testing, thyroids were dissected, fixed in 4% paraformaldehyde, dehydrated, paraffin-embedded, and sectioned at 4–5 μm. Sections were stained with hematoxylin and eosin (H&E; Solebo Biotech, Beijing, China) to assess inflammation. Lymphocytic infiltration was scored blinded as 0 (normal), 1+ (1–10%), 2+ (10–30%), 3+ (30–40%), and 4+ (≥50%). Follicular lumen and architecture were examined under a light microscope.

### 2.9 Immunofluorescence

Mice were anesthetized and transcardially perfused with cold PBS followed by 4% PFA. Tissues were cryoprotected in 30% sucrose for 2 days and sectioned at 20–40 µm. Sections were permeabilized with 0.4% Triton X-100 in PBS, blocked with 5% BSA for 1 h, and incubated overnight at 4 °C with primary antibodies against GFAP-labeled astrocytes (Cell Signaling, D1F4Q), Iba1 is a classic and widely used marker for microglia. (Abcam, ab178847), Hmgb1 (Abcam, ab190377), CD4 (Santa Cruz, sc-13573), IL-17A (Abcam, ab79056), CD68 (Abcam, ab53444), C3 (Proteintech, 66157-1-IG), and AQP4 (Santa Cruz, sc-32739). Sections were then incubated with appropriate secondary antibodies for 1 h at room temperature, and nuclei were counterstained with DAPI. Images were captured using an Olympus FV10 confocal microscope. Microglial morphology was analyzed by Sholl analysis in Fiji-ImageJ; One quantification pattern was based on DAPI, GFAP, and AQP4 immunostaining, as previously described. Briefly, in Fiji, the capillary was identified by the fluorescence-deficient lumen surrounded by DAPI- and GFAP-enriched signals, and a donut-shaped ring ROI extending 5 pixels outward from the vessel boundary was generated. AQP4 polarization was then calculated as the ratio of AQP4 fluorescence intensity within this donut ROI to the global AQP4 signal in the field: AQP4 polarization = donut-shaped area AQP4/global AQP4 (25).

### 2.10 Western blotting

Proteins were extracted from cortex and hippocampus using RIPA buffer, and concentrations were determined by BCA assay. Samples were separated by SDS-PAGE and transferred to 0.45 μm PVDF membranes. Membranes were blocked with 5% non-fat milk in TBST for 2 h, then incubated overnight at 4 °C with primary antibodies against Hmgb1 (Abcam, ab190377), IL-17A (Abcam, ab79056), and RAGE (Cell Signaling Technology, 42544; all 1:1000), with GAPDH as loading control. After incubation with HRP-conjugated secondary antibodies (1:10,000; ZSGB-BIO), bands were visualized using ECL (Meilunbio, MA0186-1) on a chemiluminescence imaging system (LI-COR Biosciences).

### 2.11 Quantitative real−time PCR

Total RNA from the hippocampus and cortex of mice was extracted using Trizol reagent (Sangon Biotech, B511311). cDNA was synthesized according to the manufacturer’s instructions using the Evo M-MLV Mix Kit with gDNA Clean (Hunan Accurate Biotechnology, AG11728). RNA purity was measured by spectrophotometry. PCR reactions were set up using SYBR^®^ Premix Ex Taq™II and amplified on the CFX96 Touch™ Real-Time PCR Detection System (Bio-Rad Laboratories, USA). Data were normalized to GAPDH expression and analyzed using the 2-ΔΔCt method.

### 2.12 Enzyme-linked immunosorbent assay

Blood samples were collected via orbital extraction under anesthesia. After standing at room temperature for 30 minutes, the samples were centrifuged at 3000 rpm for 20 minutes to obtain serum. Following euthanasia, brain tissues were rapidly harvested, homogenized, and centrifuged to collect the supernatant. Levels of mouse thyroid stimulating hormone (TSH), thyroglobulin antibody (TgAb), free thyroxine (FT4), Estradiol (E2), IL-1β (Interleukin 1 Beta), TNF-α (Tumor Necrosis Factor Alpha), IL-6 (Interleukin 6) and High mobility group proteinB1 (Hmgb1) were measured using mouse ELISA kits (Huamei Biotechnology, Bioswamp, Elabscience Biotechnology, Wuhan). All procedures were performed according to the manufacturer’s instructions.

### 2.13 Flow cytometry

Single-cell suspensions from brain tissue were prepared as previously described with minor modifications (26). Briefly, mice were anesthetized and transcardially perfused with ice-cold sterile PBS to remove intravascular residual cells. Brains were collected and placed in pre-chilled RPMI-1640 (Lonza). Tissue was mechanically dissociated and enzymatically digested with collagenase/dispase (1 mg/mL) and DNase I (10 μg/mL; Roche Diagnostics) at 37 °C for 45 min with gentle shaking (~100 rpm). The resulting cell suspension was passed through a 70-μm cell strainer and subjected to a 70%–30% Percoll density gradient to remove myelin; leukocytes were collected from the interphase and washed. Cells were blocked with anti-mouse Fc block (BioLegend) and stained with the following fluorochrome-conjugated antibodies: CD45-eFluor 450 (eBioscience, Cat# 48-0451-82), CD11b-APC (BioLegend, Cat# 101212), TMEM119-PE-Cy7 (eBioscience, Cat# 25-6119-82), and P2RY12-PE (BioLegend, Cat# 848003). Zombie Aqua (BioLegend, Cat# 423102) was used to exclude dead cells. Samples from all groups (Ctrl, EAT, and EAT+GL) were processed and stained in parallel within the same batch to minimize experimental variability. Data were acquired on a BriCyte E6 flow cytometer and analyzed in FlowJo; ≥3×10^5 events were recorded per sample. The gating strategy sequentially included FSC/SSC to exclude debris, singlets, live cells, CD45^+^ cells, and CD11b^+^ cells. CD11b^+^ cells were then separated into CD45^int and CD45^high populations based on CD45 expression, and TMEM119 and P2RY12 were assessed within each population to validate microglial identity. Positive gates were set using tissue-matched fluorescence-minus-one (FMO) and unstained controls (27, 28).

### 2.14 Statistical analysis

Fiji-ImageJ software (Version 2.9.0, NIH, Bethesda, MD, USA) was used to analyze histological and Western blot results. Statistical analyses were performed using GraphPad Prism 10.0 software. The Shapiro–Wilk test was first applied to assess data normality. For comparisons involving only two groups, an unpaired t-test was conducted. For other datasets, one-way analysis of variance (ANOVA) followed by Tukey’s multiple comparisons test was used. For the Morris water maze test, two-way repeated measures ANOVA was applied to compare the three groups. All data are presented as mean ± standard error (SE), and a p-value < 0.05 was considered statistically significant.

## 3 Results

### 3.1 Hmgb1 is actively released from hippocampal and prefrontal cortex cells in mice with autoimmune thyroiditis

Histological comparison of thyroid tissues from control (Ctrl) and experimental autoimmune thyroiditis (EAT) mice was performed both under basal conditions and after EAT induction. H&E staining confirmed the successful establishment of the EAT model, as evidenced by significant lymphocytic infiltration in the thyroid gland (**Figure 1B**). Concurrently, ELISA was performed to quantify anti-thyroglobulin antibodies (TgAb), thyroid-stimulating hormone (TSH), and free thyroxine (FT4) levels in both serum and brain tissue, confirming successful model induction while maintaining normal thyroid hormone levels (**Figure 1C**). Western blot analysis of the cerebral cortex and hippocampus revealed elevated Hmgb1 protein levels under disease conditions, suggesting a potential role for Hmgb1 in EAT-associated neuroinflammation (**Figure 1D**). Immunofluorescence (IF) staining further confirmed Hmgb1 expression in cells of the cerebral cortex and hippocampus. In Ctrl mice, Hmgb1 staining was predominantly nuclear, whereas in EAT mice, pronounced nucleocytoplasmic translocation was observed in the cortex as well as in hippocampal subregions CA1, CA3, and the dentate gyrus (DG), as indicated by yellow arrows (**Figure 1E**). Neither pTg injection nor glycyrrhizic acid administration induced any detectable change in circulating E2 levels (**Supplementary Figure S1A**). To assess systemic and CNS Hmgb1 dynamics, Hmgb1 levels were quantified by ELISA in serum, cerebrospinal fluid (CSF), and the soluble fraction of brain tissue homogenates. ELISA revealed that Hmgb1 levels were significantly elevated in EAT mice across serum, CSF, and brain soluble extracts, whereas glycyrrhizic acid (GL) treatment markedly reduced these increases (**Supplementary Figures S1B**, **S2**).

### 3.2 Inhibition of Hmgb1 reduces neurofunctional impairments and alleviates the release of inflammatory cytokines in EAT mice

**Figure 2:**
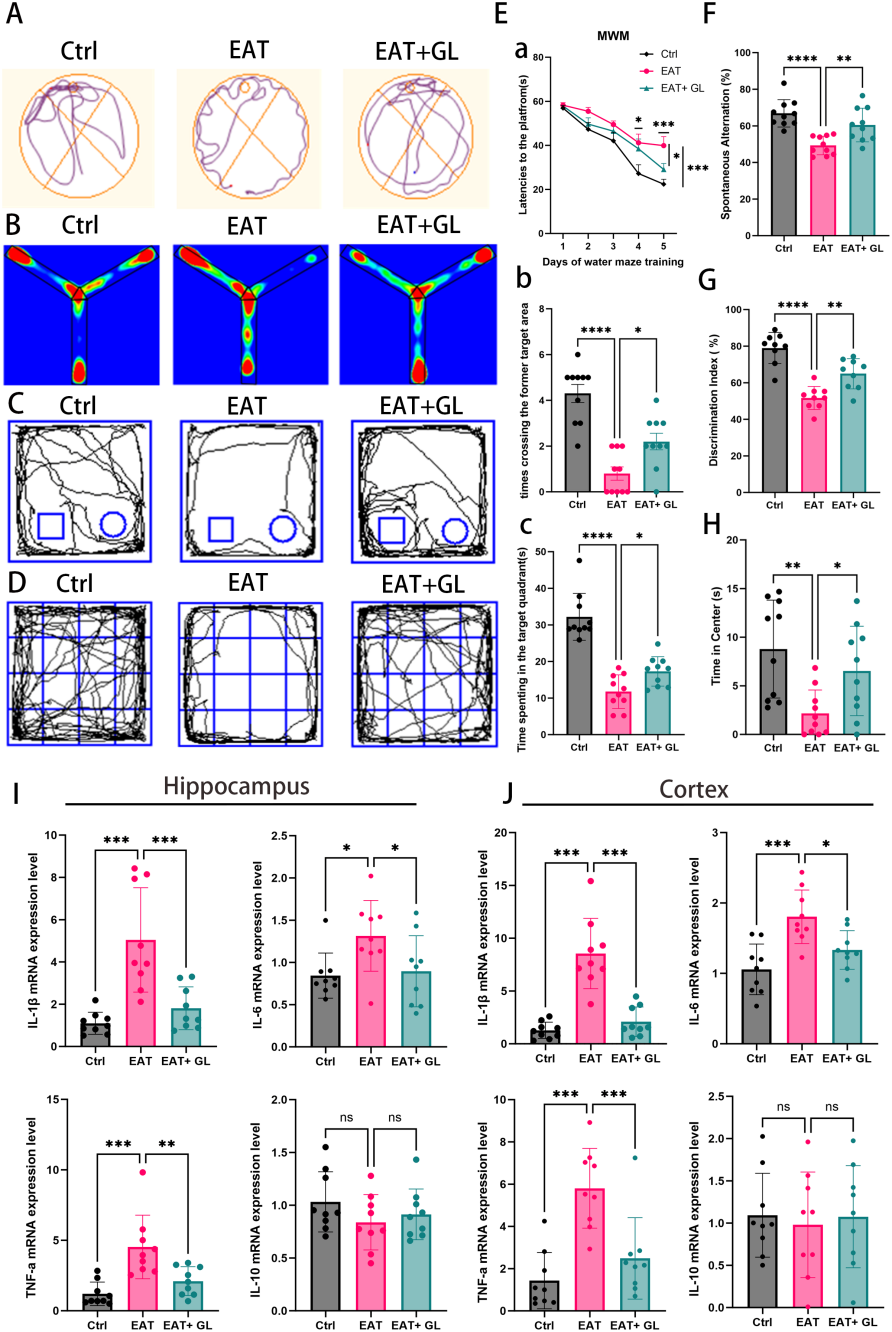
Behavioral performance and neuroinflammatory markers in EAT mice. Representative trajectories in the Morris Water Maze **(A)**, Y-maze **(B)**, novel object recognition **(C)**, and open field test **(D)**. Morris Water Maze performance **(E)**: platform latency during training **(Ea)**, time in target quadrant **(Eb)**, and number of platform crossings **(Ec)**. Y-maze spontaneous alternation percentage **(F)** and novel object recognition discrimination index **(G)**. Time spent in the center of the open field, reflecting anxiety-like behavior **(H)**. Hippocampa **(I)** and cortexl **(J)** mRNA levels of IL-1β, IL-6, IL-10, and TNF-α. Data are mean ± SD; *P ≤ 0.05; **P ≤ 0.01; ***P ≤ 0.001 (n = 10 mice pergroup).

To evaluate the functional role of Hmgb1 in EAT-induced neurobehavioral alterations, glycyrrhizic acid (50 mg/kg) was administered to EAT model mice. A battery of behavioral assays—including the Morris water maze, Y-maze, novel object recognition, and open field test—was used to assess cognitive performance and anxiety-like behaviors (**Figures 2A–D**). Concurrently, the expression of inflammatory cytokines in the hippocampus and cortex was analyzed. In the Morris water maze task, EAT mice displayed impaired spatial learning, evidenced by prolonged latency to locate the hidden platform during training. This cognitive deficit was significantly attenuated by Hmgb1 inhibition, as reflected by shorter escape latencies in the EAT+GL group on days 4 and 5 (**Figure 2Ea**). In the probe trial, the EAT+GL group spent more time in the target quadrant (**Figure 2Eb**) and showed increased platform crossing frequency (**Figure 2Ec**) compared to untreated EAT mice, indicating enhanced spatial memory retention. The Y-maze test revealed deficits in working memory in EAT mice, demonstrated by a reduced spontaneous alternation rate, which was significantly improved following Hmgb1 inhibition (**Figures 2B, F**). Similarly, the novel object recognition test showed that EAT mice exhibited impaired recognition memory, spending less time with the novel object. Treatment with glycyrrhizic acid restored novel object preference to levels comparable to control animals (**Figures 2C, G**). Anxiety-like behavior was assessed using the open field test. EAT mice spent significantly less time in the central zone, indicative of increased anxiety levels. This phenotype was reversed in the EAT+GL group, which displayed increased time in the center, consistent with reduced anxiety (**Figures 2D, H**). At the molecular level, EAT mice exhibited a significant upregulation of pro-inflammatory cytokines—including IL-1β, IL-6, and TNF-α—in both the cortex and hippocampus. These increases were markedly attenuated in the EAT+GL group. In contrast, the anti-inflammatory cytokine IL-10 showed no significant changes after Hmgb1 inhibition (**Figures 2I, J**). ELISA measurements further confirmed significantly elevated protein levels of IL-1β, IL-6 and TNF-α in EAT mice, whereas these increases were markedly reduced in the EAT+GL group (**Supplementary Figure S1C**). Collectively, these findings demonstrate that inhibition of Hmgb1 ameliorates cognitive deficits and anxiety-like behaviors in EAT mice, likely through suppression of neuroinflammation.

### 3.3 Inhibition of Hmgb1 in EAT mice reduces microglial activation

**Figure 3:**
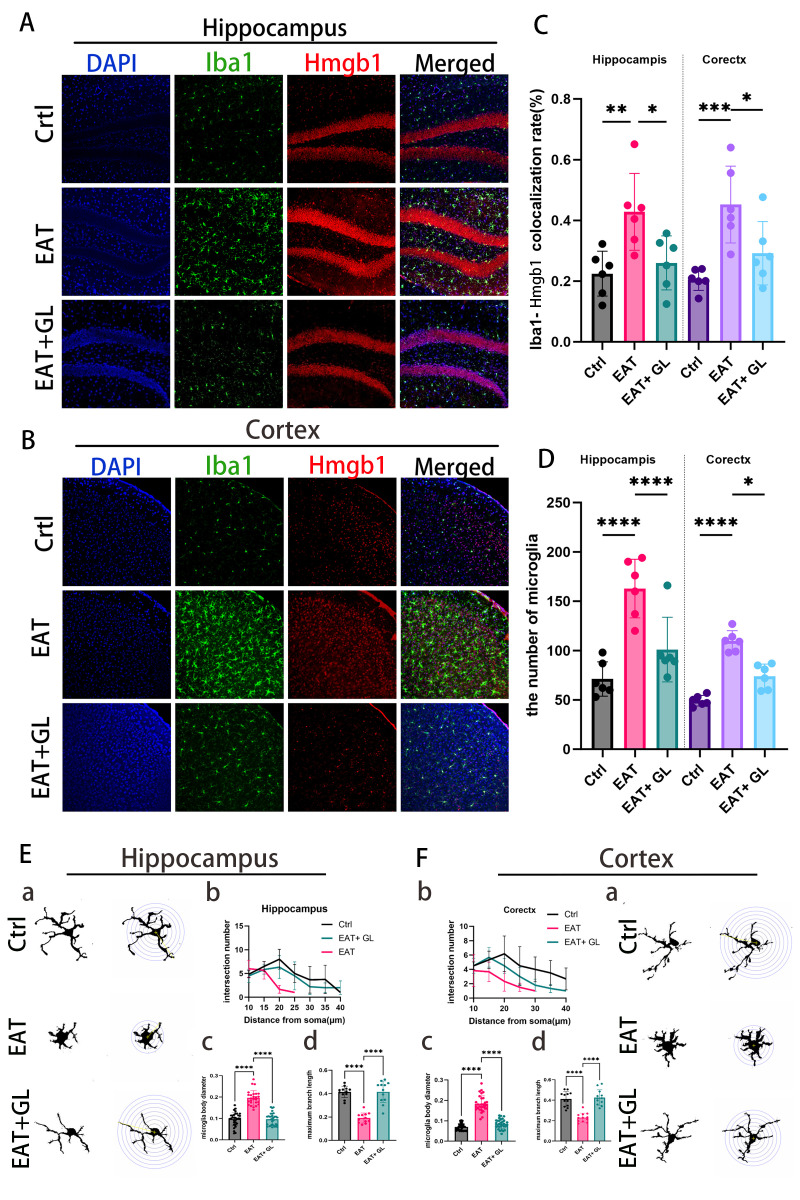
IF staining of the hippocampus showing the microglial marker Iba1 (green), Hmgb1 (red), and nuclei (DAPI, blue) at 20×magnification **(A)**. IF staining of the cortex showing Iba1 (green), Hmgb1 (red), and DAPI (blue) at 20×magnification **(B)**. Quantification of microglia co-localization with Hmgb1 in the hippocampus and cortex **(C)**. Quantification of microglial cell numbers in the hippocampus and cortex **(D)**. Representative images of Iba1 microglia in the hippocampus with Sholl analysis **(Ea)**. Line graph showing the number of intersections at increasing radial distances from the soma **(Eb)**. Quantification of cell body diameter **(Ec)**. Analysis of the number of branches per cell **(Ed)**. Representative images of Iba1 microglia in the cortex with Sholl analysis **(Fa)**. Line graph showing the number of intersections at increasing radial distances from the soma **(Fb)**. Quantification of cell body diameter **(Fc)**. Analysis of the number of branches per cell **(Fd)**. All data are presented as mean ± standard deviation (SD). *P ≤ 0.05; **P ≤ 0.01; ***P ≤ 0.001; ****P ≤ 0.0001.n = 6 mice per group.

3o investigate the effects of EAT on microglial activation and assess the impact of Hmgb1 inhibition, IF staining was performed in the cortex and hippocampus of EAT mice (**Figures 3A, B**). Quantification revealed a significant increase in Iba1 microglia in EAT mice compared to controls, which was reversed following treatment with the Hmgb1 inhibitor glycyrrhizin (**Figure 3D**). Co-labeling with anti-Iba1 and anti-Hmgb1 antibodies revealed a shift in Hmgb1 localization from the nucleus to the cytoplasm in microglia, indicating active release of Hmgb1 into the extracellular environment (**Figures 3A–C**). This suggests that microglia may serve as a source of extracellular Hmgb1 under EAT conditions. To further evaluate microglial morphology, Sholl analysis was conducted to assess process complexity and activation status (**Figures 3Ea, Fa**). EAT mice exhibited hallmark features of microglial activation, including reduced branch intersections (**Figures 3Eb, Fb**), increased soma diameter (**Figures 3Ec, Fc**), and decreased total branch number (**Figures 3Ed, Fd**). Hmgb1 inhibition attenuated these morphological changes, indicating suppression of microglial overactivation in both cortical and hippocampal regions (**Supplementary Figure S3**).

### 3.4 Hmgb1 inhibition reduces microglial activation in EAT mice

**Figure 4:**
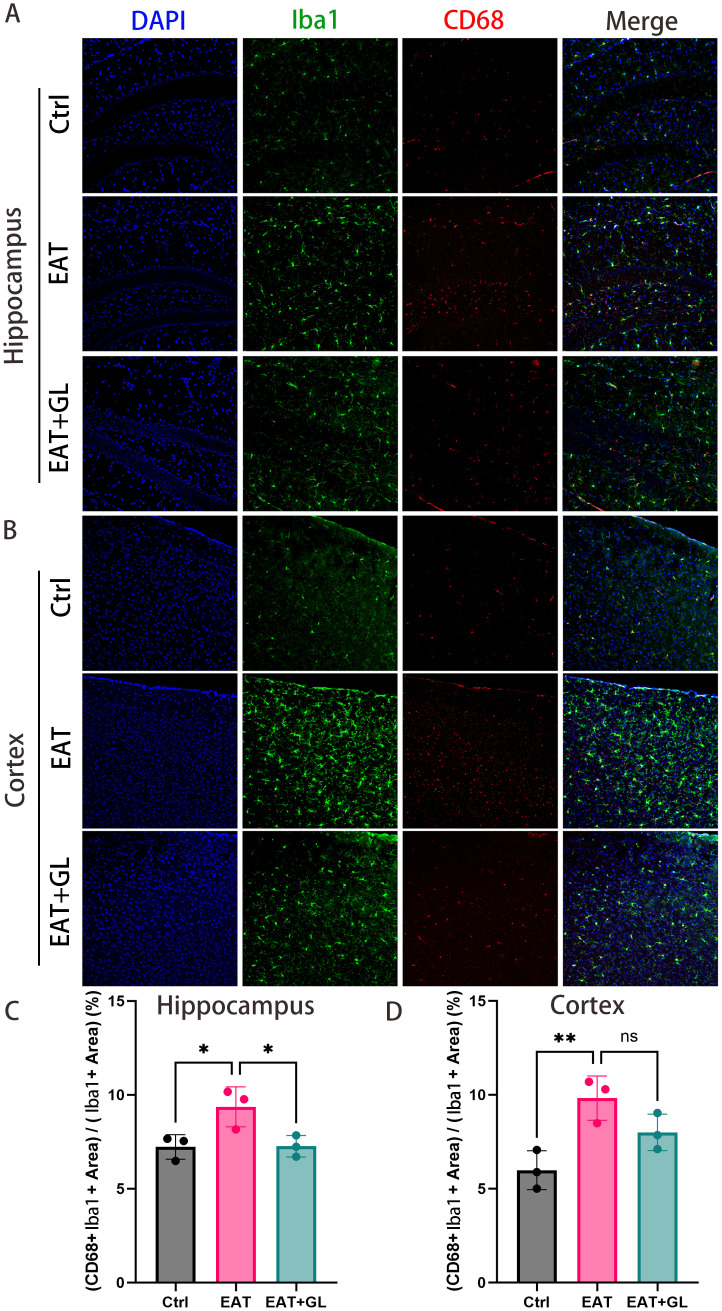
Hmgb1 inhibition reduces microglial activation in EAT mice. Representative immunofluorescence images of CD68^+^ microglia in the hippocampus **(A)** and cortex **(B)** (captured at 20× magnification), of control, EAT, and EAT+GL. Quantification of the percentage of CD68^+^Hmgb1^+^ double-positive cells relative to single-positive cells in the hippocampus **(C)** and cortex **(D)**, Data are presented as mean ± SEM; *P ≤ 0.05, **P ≤ 0.01, n = 3 mice per group.

To investigate the effect of Hmgb1 on neuroinflammation, we examined microglial activation in the hippocampus, cortex, and cerebellum of EAT mice. Immunofluorescence staining for CD68, a marker of activated microglia, revealed a marked increase in CD68 microglia in EAT mice compared with controls. Notably, treatment with an Hmgb1 inhibitor significantly reduced the proportion of microglia co-expressing CD68 in the hippocampus and cortex, indicating that Hmgb1 plays a role in microglial activation in EAT mice. (**Figure 4**) (**Supplementary Figure S4**).

### 3.5 Inhibition of Hmgb1 in EAT mice reduces astrocyte reactivity and AQP4 depolarization

**Figure 5:**
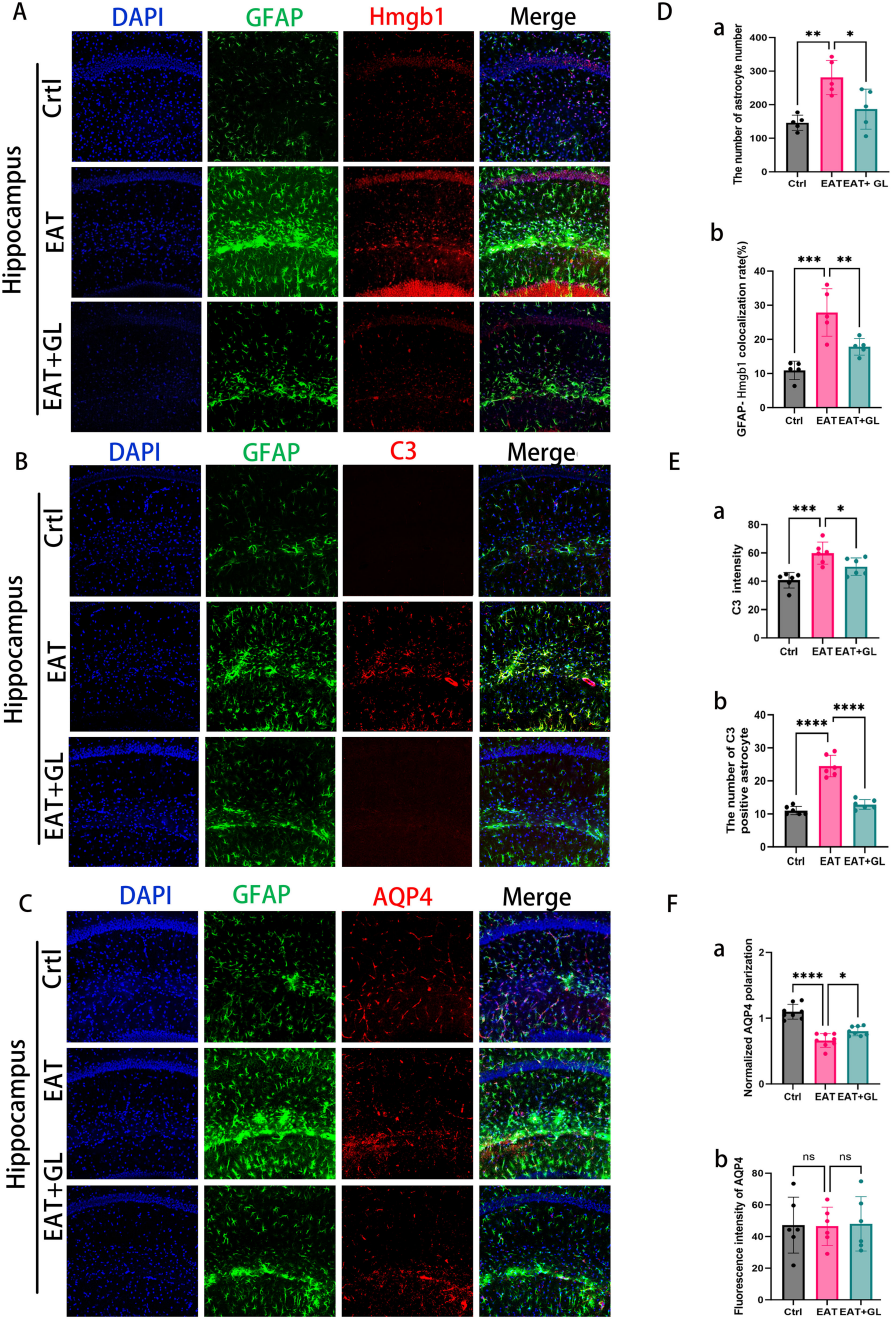
IF staining showing hippocampal GFAP (green), Hmgb1 (red), and nuclear DAPI (blue) (20× magnification), n = 5 mice **(A)**. Representative images showing expression of astrocytes (green) and AQP4 (red) **(C)**. Representative confocal microscopy images showing co-labeling of C3 (red, an A1 marker) and GFAP (green) **(B)**. Quantification of astrocyte number **(Da)** and co-localization rate of astrocytes with Hmgb1 **(Db)**. C3 immunofluorescence intensity **(Ea)** and number of C3-positive (A1-like) astrocytes **(Eb)**. AQP4 polarity **(Fa)** and AQP4 expression intensity **(Fb)**. Each dataset is presented as mean ± standard deviation. *P ≤ 0.05; **P ≤ 0.01; ***P ≤ 0.001; ****P ≤ 0.0001, n = 6 mice.

To further characterize the role of astrocytes in HT-associated neuroinflammation, we investigated the impact of EAT on hippocampal astrocyte reactivity and the modulatory effects of Hmgb1 inhibition. IF analysis revealed a marked increase in GFAP^+^ astrocyte density in the hippocampus of EAT mice, which was significantly reduced following Hmgb1 inhibition (**Figures 5A, Da**). Co-localization of GFAP and Hmgb1 demonstrated nuclear-to-cytoplasmic translocation of Hmgb1 in astrocytes, suggesting that astrocytes may serve as an additional source of extracellular Hmgb1 during HT-related neuroinflammation (**Figure 5Db**). To assess astrocyte phenotypic changes, IF staining for GFAP and complement component 3 (C3) was performed. Compared to controls, EAT mice showed elevated C3 expression, indicative of a shift toward the neurotoxic A1 astrocyte phenotype. Notably, Hmgb1 inhibition reduced C3 expression and attenuated A1 polarization (**Figures 5B, Ea, b**), suggesting a role for Hmgb1 in astrocyte-driven complement activation and inflammatory signaling. Using GFAP and AQP4 double staining with quantification via the Donut method (25), we observed a significant loss of AQP4 polarity in EAT mice relative to controls, indicative of glymphatic disruption. Strikingly, Hmgb1 inhibition restored AQP4 polarity, further supporting its therapeutic potential in preserving astrocytic function and CNS fluid dynamics (**Figures 5C, Fa, b**) (**Supplementary Figure S5**).

### 3.6 In EAT mice, peripheral CD4^+^ T cells infiltrate and release IL-17A, and inhibition of Hmgb1 reduces IL-17A release

**Figure 6:**
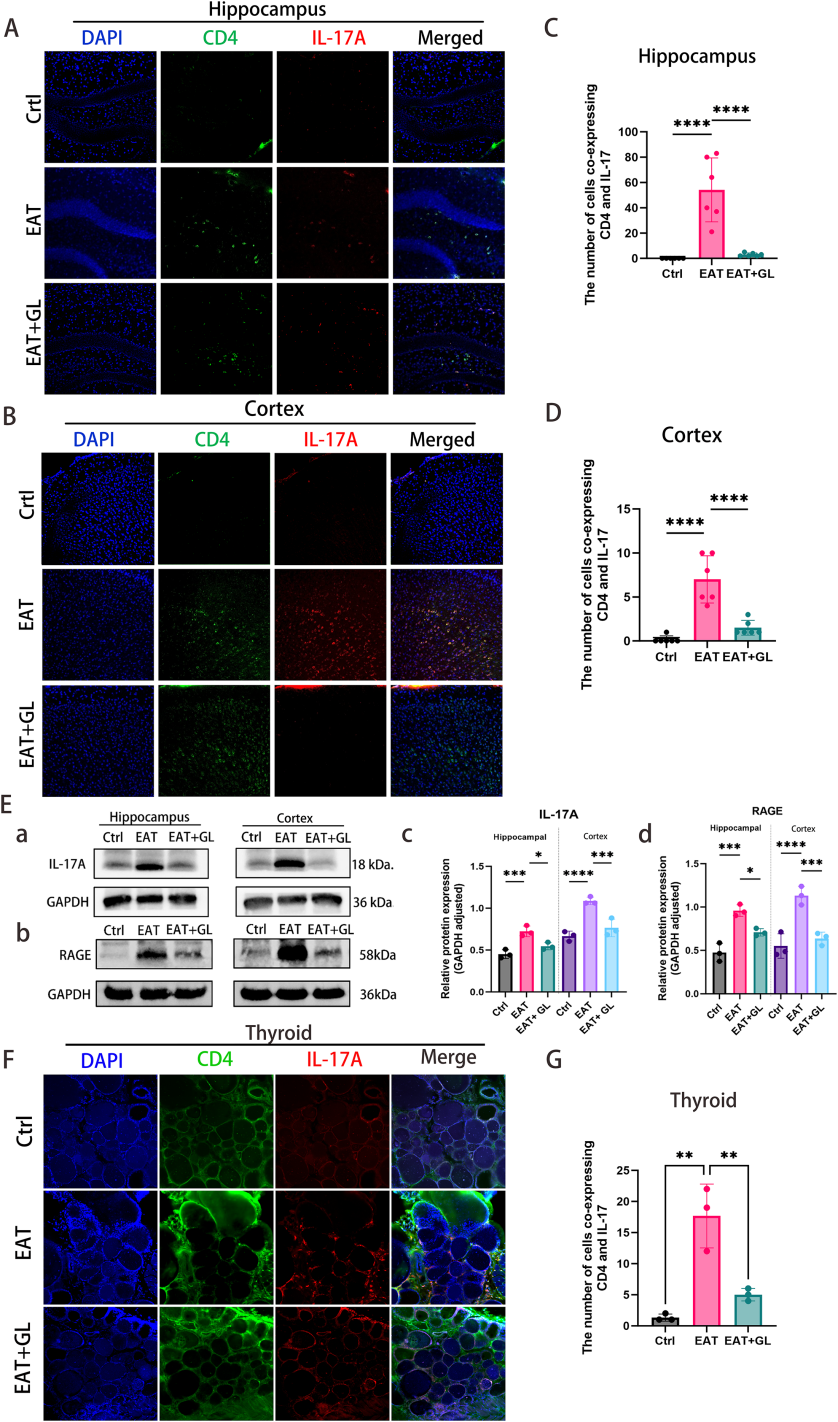
CD4^+^ T cells and IL-17A infiltration and protein expression in hippocampus, cortex, and thyroid. IF staining of hippocampus **(A)** and cortex **(B)** showing CD4 (green), IL-17A (red), DAPI (blue), and merged signals. Quantification of co-localized CD4^+^ T cells and IL-17A in hippocampus **(C)** and cortex **(D)**. Western blot analysis of IL-17A, Hmgb1 and RAGE protein levels in hippocampus and cortex (n = 3 mice) **(E)**. IF staining of thyroid showing CD4 (green), IL-17A (red), DAPI (blue), and merged signals **(F)**. Quantification of co-localized CD4^+^ T cells and IL-17A in thyroid (n = 3 mice) **(G)**. Data are presented as mean ± SD. *P ≤ 0.05; **P ≤ 0.01; ***P ≤ 0.001; ****P ≤ 0.0001 (n = 6 mice).

To investigate the involvement of CD4^+^ T cells and IL-17A in EAT-induced CNS injury, we performed IF and Western blot analysis. IF staining revealed a marked accumulation of CD4^+^ T cells in the cortex and hippocampus of EAT mice, with a greater density observed in the cortical region. These infiltrating CD4^+^ T cells exhibited robust IL-17A expression (**Figures 6A–D**). Hmgb1 inhibition significantly reduced IL-17A production by CD4^+^ T cells, as confirmed by IF co-localization (**Figures 6C, D**) and Western blotting (**Figures 6Ea, Ec**). In parallel, expression of the receptor for advanced glycation end products (RAGE)—a key surface receptor mediating Hmgb1 signaling—was elevated in EAT mice but was down regulated following Hmgb1 blockade (**Figures 6Eb, di**). Inhibition of Hmgb1 effectively suppressed IL-17A release in the thyroid, suggesting that Hmgb1 contributes to local Th17-mediated inflammation (**Figure 6G**) (**Supplementary Figure S6**).

### 3.7 EAT increases infiltrating myeloid cells in the brain, which is partially reversed by GL treatment

To distinguish resident microglia from infiltrating peripheral myeloid cells, we performed flow cytometry on brain single-cell suspensions. Within CD45^+^CD11b^+^ myeloid cells, we separated CD45^int and CD45^high subsets based on CD45 intensity and further assessed TMEM119 and P2RY12 within each subset to validate microglial identity. Compared with controls, the EAT group showed a marked increase in the CD11b^+^CD45^high population (Ctrl: 3.12% vs EAT: 12.4%), accompanied by a decrease in the CD11b^+^CD45^int population (Ctrl: 83.5% vs EAT: 67.2%). These changes were partially restored by GL treatment (EAT+GL: CD45^high 5.08%, CD45^int 76.9%). Consistent with a microglial phenotype, the majority of cells within the CD11b^+^CD45^int gate were TMEM119^+^P2RY12^+^ across groups (Ctrl: 97.4%, EAT: 91.0%, EAT+GL: 94.5%). In contrast, TMEM119^+^P2RY12^+^ events were generally low within the CD11b^+^CD45^high gate (Ctrl: 5.65%, EAT+GL: 4.02%), indicating that this population is enriched for infiltrating myeloid cells. Notably, the TMEM119^+^P2RY12^+^ fraction within CD45^high increased to 18.0% in the EAT group, which may reflect inflammation-associated CD45 upregulation in activated microglia leading to partial overlap with the CD45^high gate. Collectively, these data indicate that EAT promotes accumulation of CD45^high infiltrating myeloid cells in the brain, whereas GL treatment reduces this infiltrating compartment and partially normalizes the distribution of microglia-associated populations (**Supplementary Figure S7**).

## 4 Discussion

Accumulating evidence suggests that HT extends beyond a purely organ-specific pathology, predisposing patients to neuropsychiatric comorbidities such as depression. The pathways linking peripheral immune dysregulation to central dysfunction, however, are still unclear. Our findings identify that chronic inflammation triggers aberrant neuronal activity and propagates a self-amplifying circuit of CD4^+^ T cell infiltration and IL-17A release, which collectively drives progressive neuroinflammation. Of pivotal importance, targeting Hmgb1 broke this circuit, resulting in suppressed neuroinflammation and rescued behavioral deficits, thereby affirming its role as a key node with therapeutic implications (29–31).

Under normal physiological conditions, the intact BBB restricts the entry of circulating antibodies into the CNS (32). However, in HT, multiple factors may facilitate antibody translocation across the BBB. Proinflammatory cytokines such as TNF-α and IL-6 have been shown to increase BBB permeability by downregulating tight junction proteins, including claudin-5 and occludin. Furthermore, thyroid autoantigens—thyroid-stimulating hormone receptor (TSH-R), thyroglobulin (Tg) and thyroid peroxidase (TPO)—exhibit substantial structural homology with various CNS proteins. This molecular mimicry may lead to cross-reactivity between thyroid autoantibodies and CNS antigens, potentially contributing to neurological manifestations in HT patients (33). Once inside the CNS, anti-TPO and anti-Tg antibodies may bind directly to hippocampal neurons or glial cells—possibly via cross-reactivity with neuronal enolase—thereby promoting neuroinflammatory responses (34, 35). Immunofluorescence analyses in EAT model mice revealed pronounced microglial activation with amoeboid, phagocytic morphology, accompanied by reactive astrogliosis evidenced by GFAP upregulation. These findings support the concept of antibody-driven neuroinflammation in HT (36).

Hmgb1 is a highly conserved nuclear protein involved in DNA transcription, recombination and repair under physiological conditions. In pathological contexts, Hmgb1 is released extracellularly via two routes: passive leakage from necrotic cells and active secretion from immune cells—such as macrophages and microglia—via non-classical secretory pathways upon inflammatory stimulation (37–39). Under physiological conditions, Hmgb1 resides in the nucleus; however, upon activation, it translocates to the cytoplasm or extracellular space, where it is functions as a cytokine or chemokine to initiate inflammatory signaling—a hallmark of Hmgb1 activation (40, 41). Elevated Hmgb1 levels have been implicated in the pathogenesis of multiple neurodegenerative disorders (42). In the EAT mouse model, we observed robust activation of microglia and astrocytes in the hippocampus and prefrontal cortex. This glial response was accompanied by Hmgb1 translocation from the nucleus to the cytoplasm, together with increased fluid-phase Hmgb1 levels in serum and cerebrospinal fluid (CSF), supporting enhanced release-associated Hmgb1 under EAT conditions. Together, these results indicate that Hmgb1 is a key driver of EAT-induced microglial activation, and that inhibiting Hmgb1 effectively counteracts the structural and numerical alterations characteristic of neuroinflammation (43).

This observation is consistent with clinical findings of elevated Hmgb1 levels in patients with HT, suggesting that peripheral–central immune crosstalk in HT may drive glial activation via Hmgb1 signaling (21). Furthermore, Hmgb1 accumulation was positively correlated with increased levels of proinflammatory cytokines, including TNF-α, IL-1β, and IL-6. Importantly, administration of glycyrrhizin, a selective Hmgb1 inhibitor, significantly reduced the expression of proinflammatory cytokines while enhancing the production of the anti-inflammatory cytokine IL-10. It also suppressed M1-type microglial polarization and reduced complement C3 secretion from astrocytes, thereby disrupting the self-perpetuating inflammatory loop. These results align with previous findings in other neuroinflammatory conditions, such as multiple sclerosis, and support a central role for Hmgb1 in HT-associated neuroinflammation (44). Notably, we also report for the first time that HT alters astrocytic aquaporin-4 (AQP4) polarization. AQP4 is essential for cerebral water homeostasis, and its polarized expression at astrocytic endfeet near blood vessels is critical for brain–cerebrospinal fluid barrier function (45). In HT model mice, AQP4 distribution was disrupted, characterized by reduced perivascular expression and increased localization in astrocytic somata and processes. Hmgb1 inhibition not only attenuated glial activation but also ameliorated cognitive and behavioral deficits, as demonstrated by improved performance in the Morris water maze, novel object recognition, and Y-maze tasks, as well as reduced anxiety-like behavior in the open field test. These findings highlight the therapeutic potential of targeting Hmgb1 in HT-associated neuropsychiatric symptoms.

Glycyrrhizin and its active metabolites have been reported to inhibit 11β-hydroxysteroid dehydrogenase type 2 (11β-HSD2), thereby reducing glucocorticoid inactivation and potentially altering local or systemic glucocorticoid exposure (46). Notably, the distribution of 11β-HSD isoforms differs between peripheral tissues and the adult CNS: 11β-HSD1 is more widely expressed in the adult brain and contributes to local regeneration of active glucocorticoids, whereas 11β-HSD2 expression in the adult brain is relatively limited and is more prominent during development (47). Thus, whether 11β-HSD2 inhibition by glycyrrhizin directly drives substantial CNS glucocorticoid changes and behavioral outcomes in adult animals should be interpreted with caution. In our study, the anxiolytic-like effects of glycyrrhizin are more plausibly linked to its anti-inflammatory actions in the CNS and prior studies have shown that inhibition of Hmgb1 or Hmgb1/RAGE signaling alleviates neuroinflammation-associated depressive- and anxiety-like phenotypes (48, 49).

While prior work has shown that the EAT model disrupts the BBB and highlighted the central role of CD4^+^ T cells in thyroid autoimmunity, the specific mechanisms linking peripheral immunity to CNS inflammation remain poorly defined. It is known that Treg dysfunction and Th1/Th17 imbalances, especially the production of IL-17A, contribute to autoimmune pathology and neuroinflammation (50). CD4^+^ T cells, as key adaptive immune regulators, play complex roles in the CNS—driving neuroinflammation in conditions like MS via Th1/Th17 infiltration and cytokine release (51, 52), and modulating pathology in neurodegenerative diseases such as AD and PD, where certain subsets may exert protection (53, 54).

Critically, we demonstrate for the first time the infiltration of CD4^+^ T cells into the cerebral cortex and hippocampus of HT model mice, with a significant portion producing IL-17A. This offers direct evidence of peripheral immune cell trafficking into the CNS in HT, likely via disrupted BBB integrity or meningeal lymphatic vessels. Together with our behavioral data, these findings support a model wherein Hmgb1–RAGE signaling facilitates CD4^+^ T cell entry into the brain parenchyma, leading to local IL-17A secretion and the exacerbation of neuroinflammatory injury. Flow cytometry using CD11b/CD45 gating with TMEM119 and P2RY12 showed that CD11b^+^CD45^int cells were consistently TMEM119^+^P2RY12^+^ microglia, whereas CD11b^+^CD45^high cells had low TMEM119/P2RY12 and were annotated as an infiltrating myeloid–enriched compartment. The CD11b^+^CD45^high fraction increased in EAT and was reduced by glycyrrhizin toward control levels, and together with cortical/hippocampal CD4^+^ (including IL-17A^+^) T-cell infiltration, supports peripheral immune trafficking as a contributor to CNS neuroinflammation in this model.

Several aspects of this work warrant further consideration. The EAT model, while instrumental, cannot fully replicate the complex chronicity of human HT. Moreover, the mechanisms driving CD4^+^ T cell infiltration, including the contribution of specific chemokine networks, require further definition. Translating our findings, future research should explore Hmgb1 and IL-17A as potential biomarkers in the cerebrospinal fluid of HT patients and examine their correlation with clinical neuropsychiatric manifestations. A deeper understanding of the Th17/Treg balance will also be pivotal in unraveling the immunoregulatory dysfunction in HT-associated neuroinflammation. Future studies will prioritize incorporating estrous cycle staging together with longitudinal measurements of sex steroids. The estrous phase will be accounted for as a covariate and used for stratified analyses to more rigorously control for potential influences of sex hormones and cycle stage on behavioral phenotypes. Our findings primarily support an Hmgb1-driven neuroinflammatory and functional impairment model in EAT mice, and future studies will be needed to determine whether overt neurodegenerative changes occur.

## 5 Conclusion

**Figure 7:**
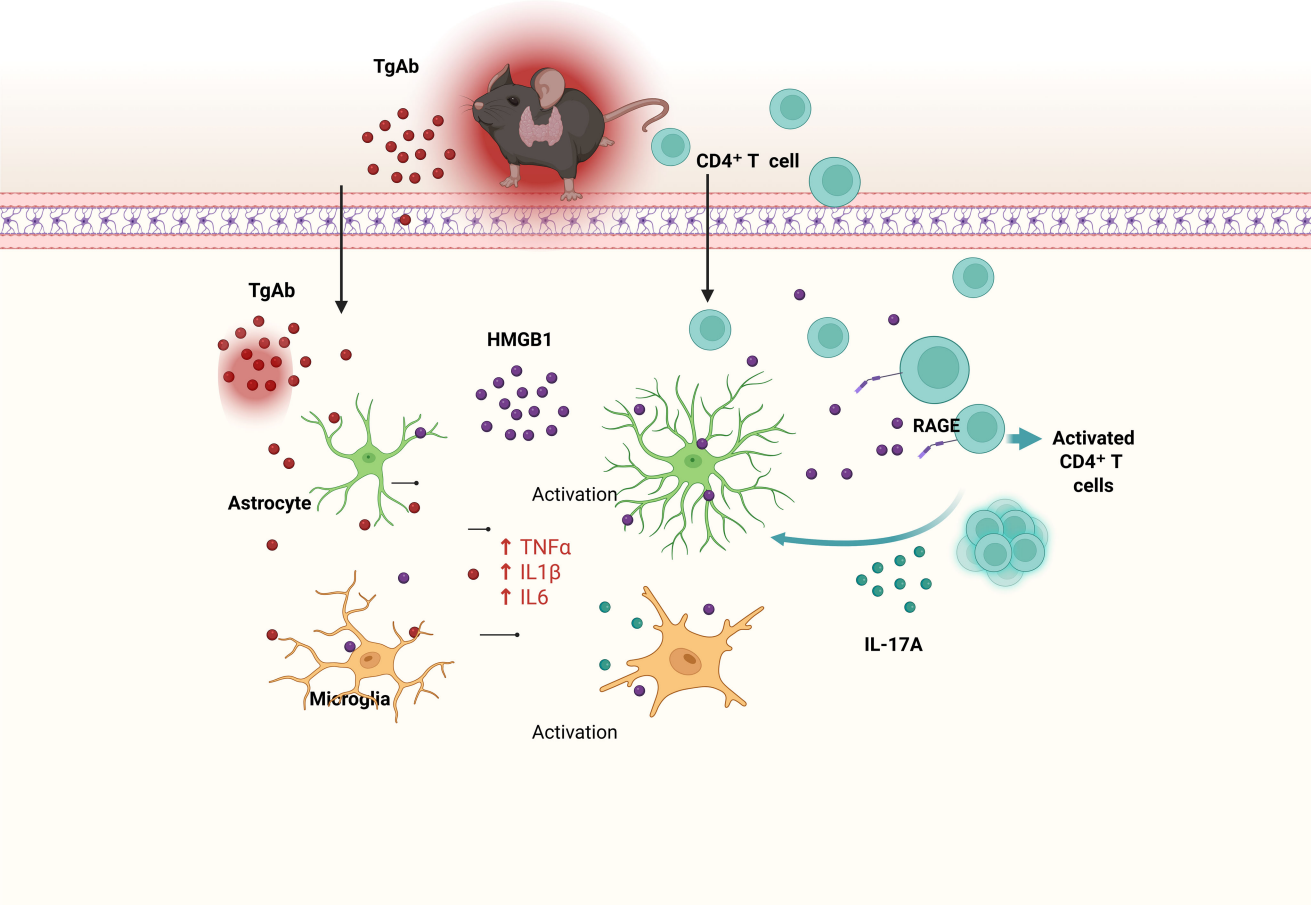
Here we propose a mechanistic model linking experimental autoimmune thyroiditis (EAT) to neuroinflammation and cognitive deficits. EAT triggers peripheral immune activation, characterized by elevated levels of thyroglobulin antibodies (TgAb) and recruitment of CD4^+^ T cells. TgAb and infiltrating CD4^+^ T cells cross the blood–brain barrier (BBB) and enter the brain, where they activate microglia and astrocytes. Activated glial cells release extracellular high-mobility group box 1 (HMGB1), which further stimulates CD4^+^ T-cell activation via the receptor for advanced glycation end products (RAGE), leading to increased IL-17A production. IL-17A, in turn, amplifies microglial and astrocytic activation and upregulates pro-inflammatory cytokines such as TNF-α, IL-1β, and IL-6. This cascade exacerbates neuroinflammation and ultimately contributes to cognitive impairment. Key: TgAb (red dots), HMGB1 (purple dots), CD4^+^ T cells (cyan circles), IL-17A (cyan small dots), astrocytes (green), microglia (orange); solid arrows indicate promoting effects. Created in BioRender. Q, Q. (2026) https://BioRender.com/6ff1fxp.

Our research delineates a previously unknown pathway through which thyroid autoimmunity drives CNS inflammation. We provide evidence that thyroid autoantibodies translocate across the BBB, instigating an Hmgb1-dependent activation of glial cells. This key event initiates a multistep inflammatory cascade: it promotes CD4^+^ T cell recruitment into the CNS, enhances antigen presentation, and skews T cell differentiation toward the Th17 lineage, thereby boosting IL-17A production. This cascade collectively exacerbates neurotoxic responses and perpetuates chronic neuroinflammation, identifying the pathway as a viable target for treating HT-associated CNS complications (**Figure 7**).

## Data Availability

The raw data supporting the conclusions of this article will be made available by the authors, without undue reservation.
