# Supplementary material for: HMGB1 dysregulation: a neuroimmune bridge to cognitive impairment in autoimmune thyroiditis
Source: Front Immunol. 2026 Feb 17;17:1764288. doi: 10.3389/fimmu.2026.1764288 (PMC12953525; doi:10.3389/fimmu.2026.1764288)
Supplement: Supplementary file 1 [file DataSheet1.pdf]

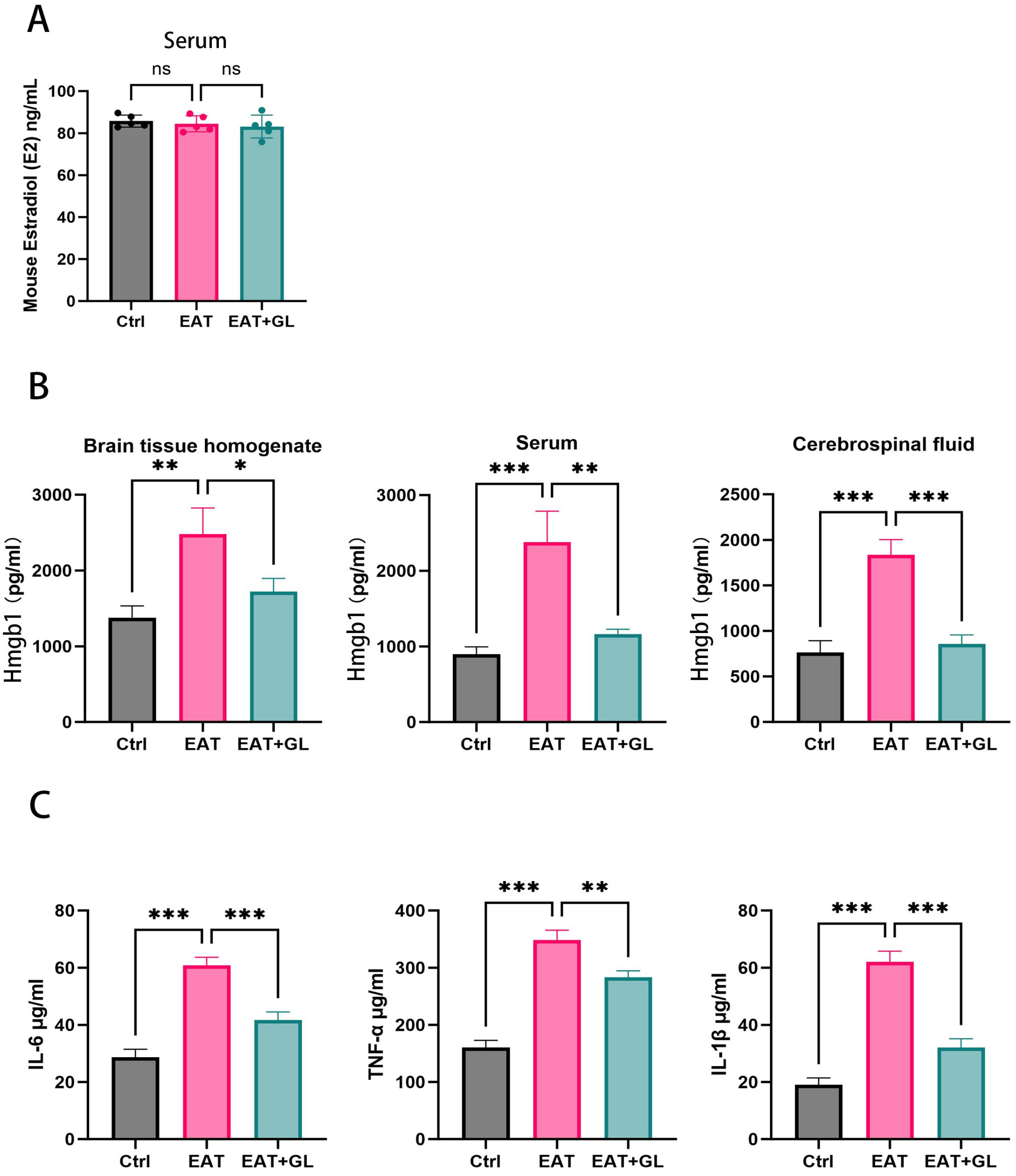

Figure. S1 EAT elevates Hmgb1 and pro-inflammatory cytokines without altering serum estradiol levels; GL attenuates the inflammatory responses.

Serum estradiol (E2) levels (ng/mL) showing no significant differences among groups (ns) (A). Hmgb1 levels (pg/mL) in brain tissue homogenates, serum, and cerebrospinal fluid (B). Brain tissue homogenates levels of IL-6, TNF-α, and IL-1β (units as indicated) (C). Data are presented as mean ± SEM, with each dot representing one biological replicate. Statistical analyses were performed using one-way ANOVA followed by Tukey's multiple comparisons test. Brackets indicate pairwise comparisons. $*P < 0.05$, $**P < 0.01$, $***P < 0.001$; ns, not significant. n =3 per group.

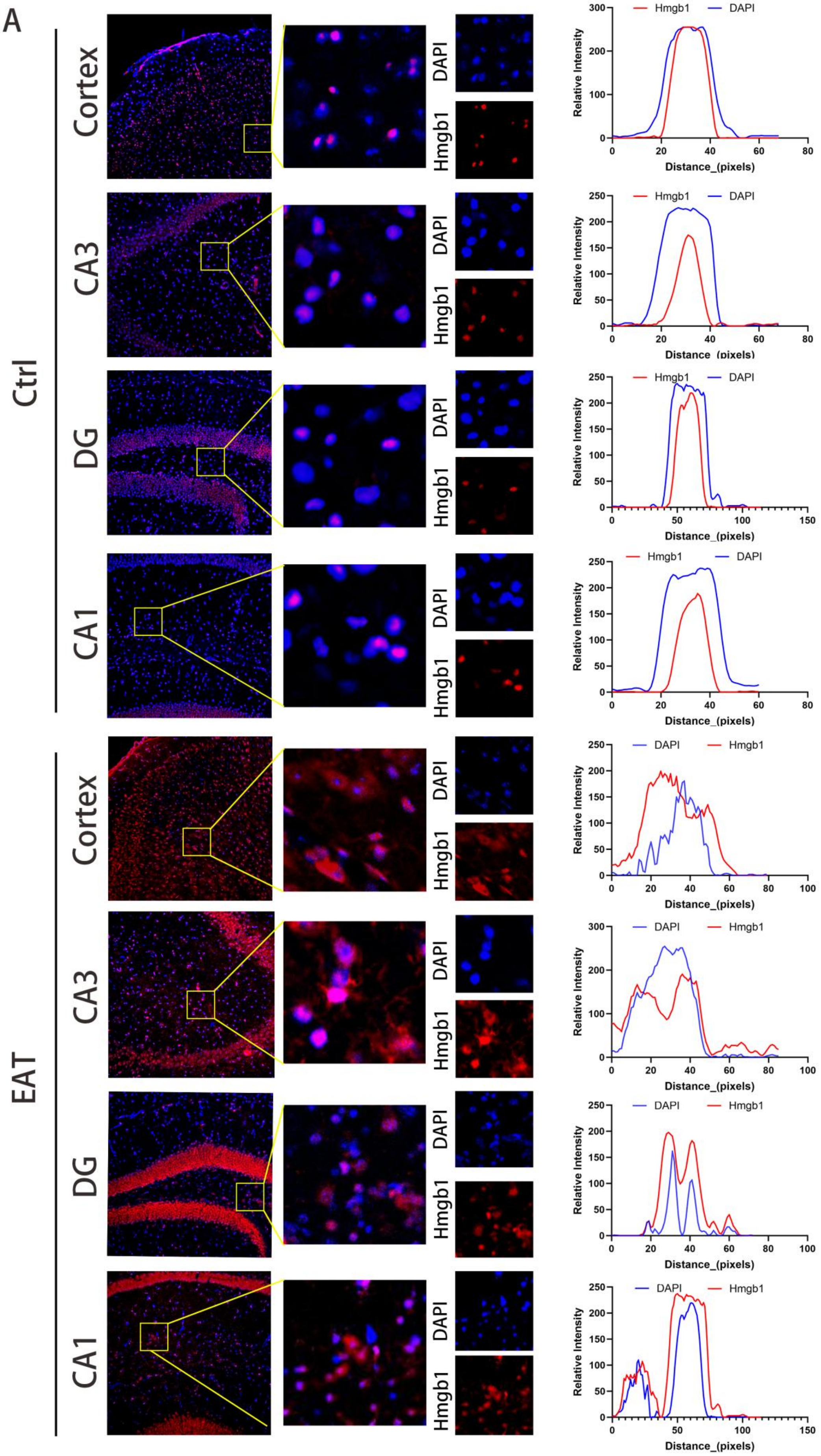

A
Ctrl
EAT
Cortex
CA3
DG
CA1
DAPI
Hmgb1
Relative Intensity
Distance_(pixels)

Figure. S2 Representative immunofluorescence images and line-scan analyses show Hmgb1 redistribution from the nucleus in the EAT group.

Brain sections from Ctrl and EAT mice were stained for Hmgb1 (red) with DAPI (blue) marking nuclei. Left panels show low-magnification images with yellow boxes indicating regions of interest; middle panels show corresponding high-magnification views. Right panels display single-channel images and fluorescence intensity line profiles (line-scan) for Hmgb1 and DAPI. In Ctrl, Hmgb1 largely overlaps with DAPI, indicating predominant nuclear localization, whereas in EAT, reduced overlap and increased extranuclear Hmgb1 signal suggest nuclear-to-cytoplasmic/extracellular translocation. Regions shown include cortex, hippocampal CA3, dentate gyrus (DG), and CA1.

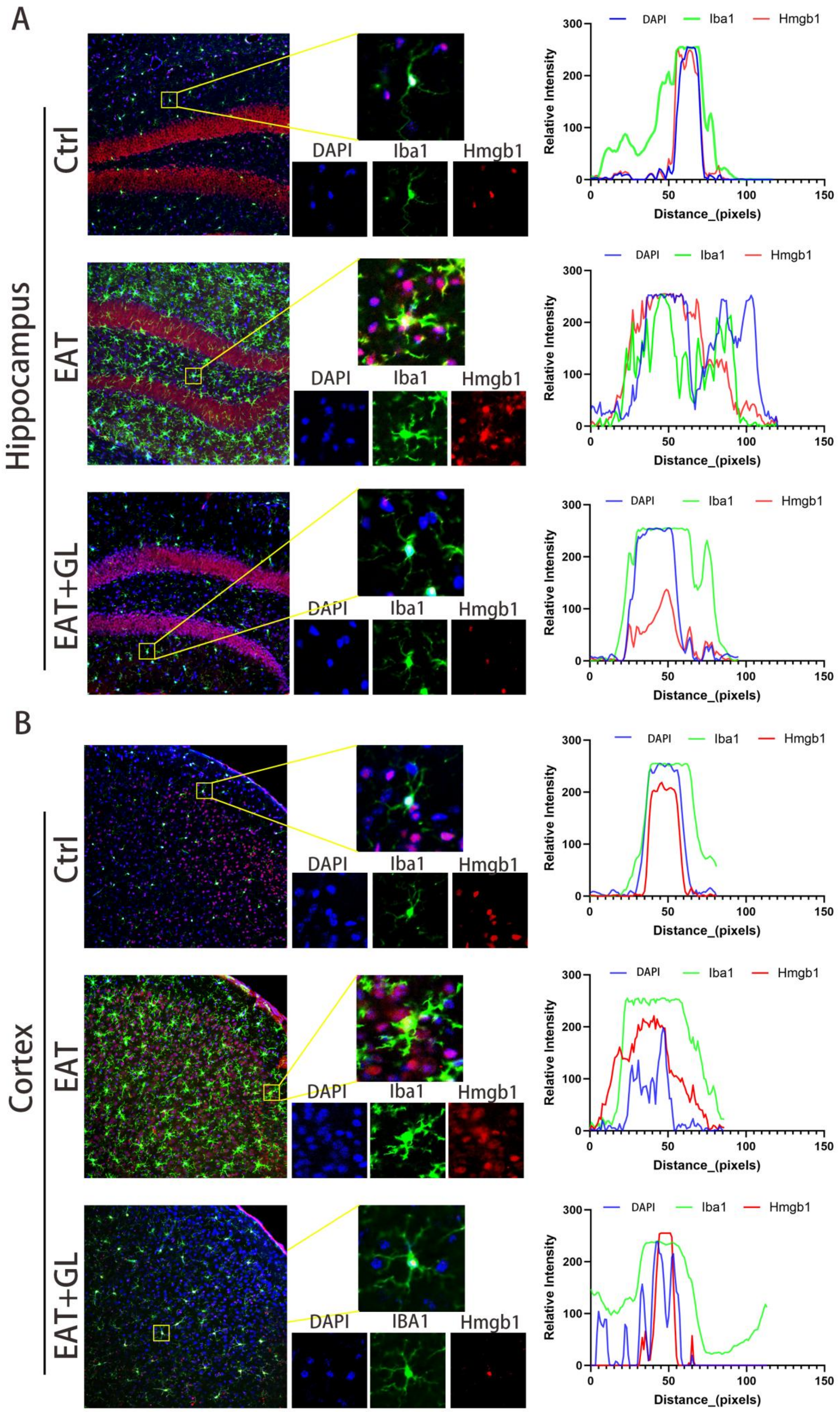

A
Hippocampus
Ctrl
EAT
EAT+GL
DAPI
Iba1
Hmgb1
Relative Intensity
Distance_(pixels)
B
Cortex
IBA1

Figure. S3 Immunofluorescence staining and line-scan analyses of Iba1 and Hmgb1 in the hippocampus and cortex.

Brain sections from Ctrl, EAT, and EAT+GL mice were stained for Iba1 (green), Hmgb1 (red), and DAPI (blue). Representative images from the hippocampus (A); representative images from the cortex (B). Left panels show low-magnification views with yellow boxes indicating regions of interest; middle panels show corresponding high-magnification images and single-channel signals. Right panels show fluorescence intensity line profiles (line-scan) for DAPI, Iba1, and Hmgb1, illustrating their spatial distribution.

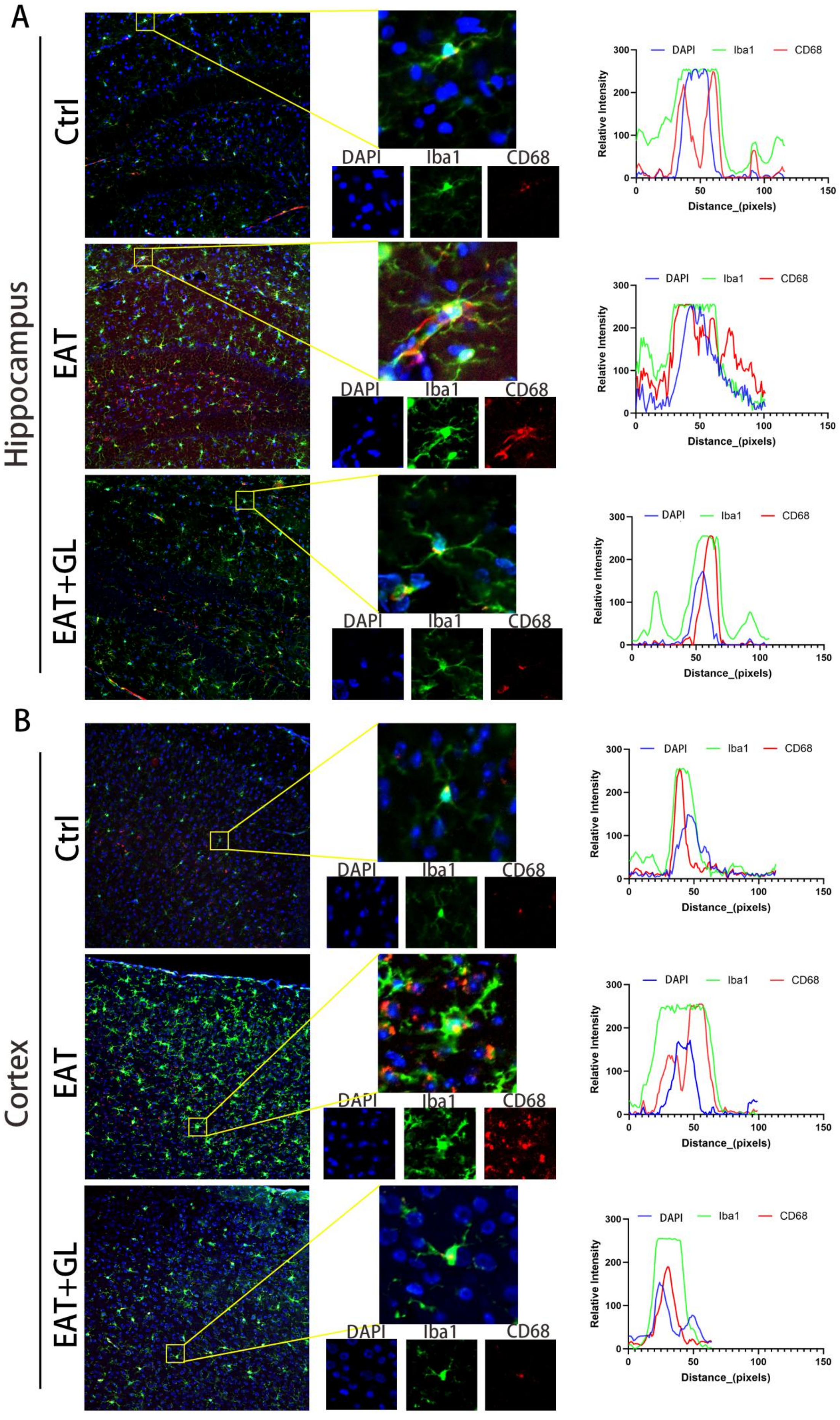

A
Hippocampus
Ctrl
EAT
EAT+GL
DAPI
Iba1
CD68
Relative Intensity
Distance_(pixels)
B
Cortex

Figure. S4 Immunofluorescence staining and line-scan analyses of Iba1 and CD68 in the hippocampus and cortex.

Brain sections from Ctrl, EAT, and EAT+GL mice were stained for Iba1 (green), CD68 (red), and DAPI (blue). Representative images from the hippocampus (A); representative images from the cortex (B). Left panels show low-magnification views with yellow boxes indicating regions of interest; middle panels show corresponding high-magnification images and single-channel signals. Right panels show fluorescence intensity line profiles (line-scan) for DAPI, Iba1, and CD68, illustrating their spatial distribution.

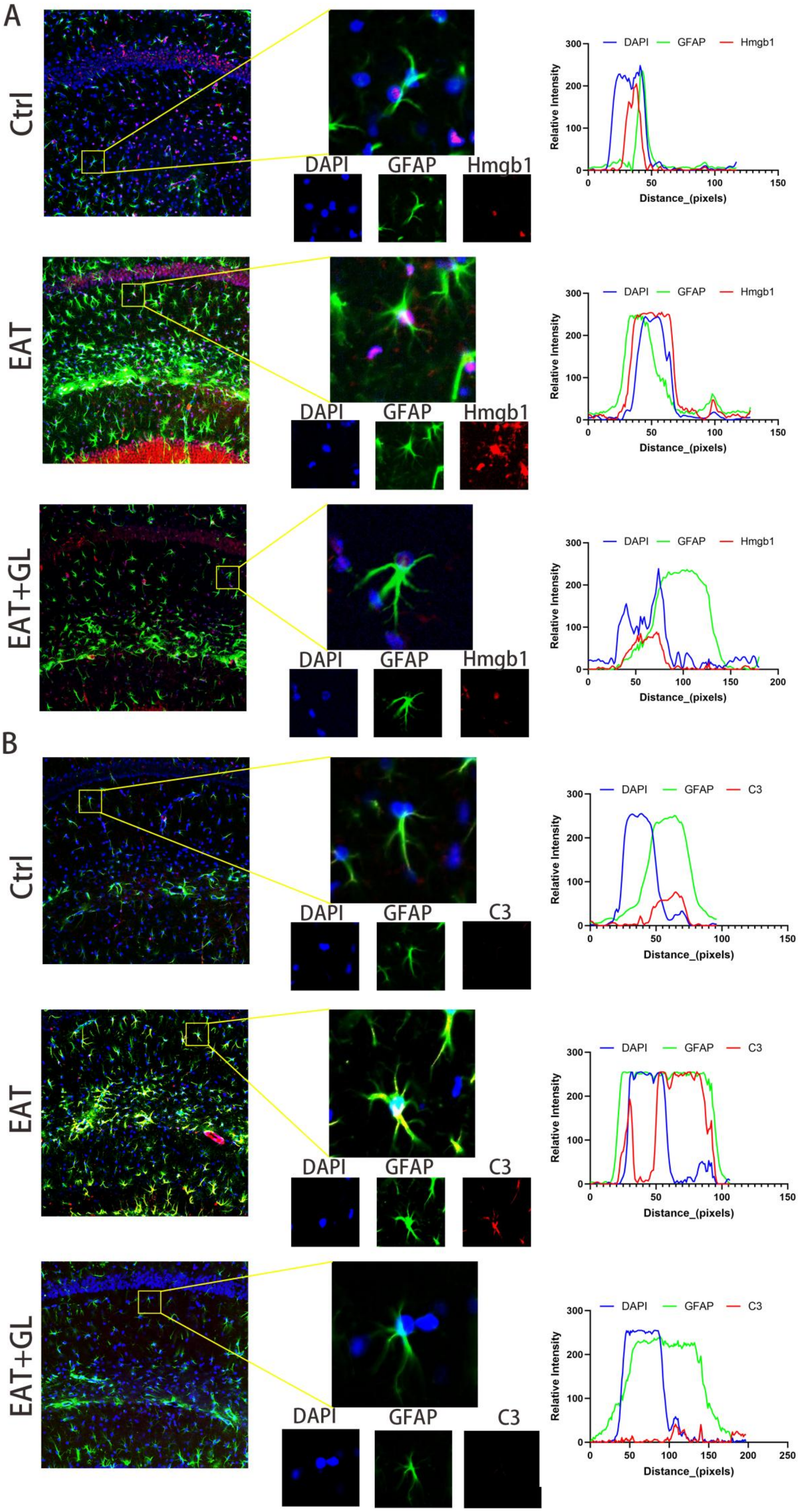

A
Ctrl
EAT
EAT+GL
DAPI
GFAP
Hmgb1
Relative Intensity
Distance_(pixels)
B
C3

Figure. S5 Immunofluorescence staining and line-scan analyses of astrocytic GFAP with Hmgb1 or C3.

Brain sections from Ctrl, EAT, and EAT+GL mice were stained for GFAP (green) and DAPI (blue). Co-staining for Hmgb1 (red) (A). Co-staining for C3 (red) (B). Left panels show low-magnification representative images with yellow boxes indicating regions of interest; middle panels show corresponding high-magnification views and single-channel images. Right panels show fluorescence intensity line profiles (line-scan) for DAPI, GFAP, and Hmgb1/C3, illustrating their spatial distribution and proximity.

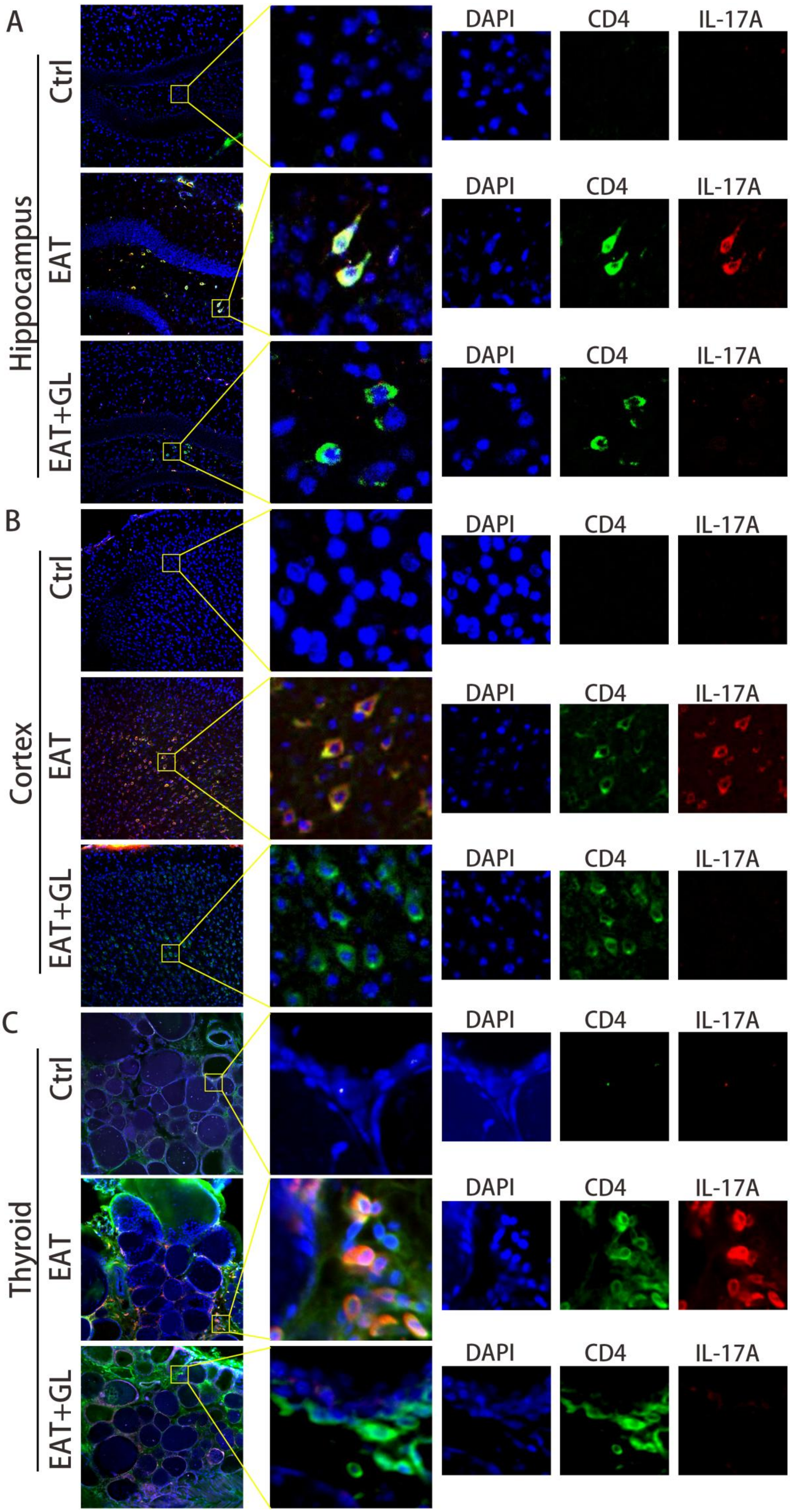
A
B
C
Hippocampus
Cortex
Thyroid
Ctrl
EAT
EAT+GL
DAPI
CD4
IL-17A

Figure. S6 Immunofluorescence staining of CD4 and IL-17A in the hippocampus, cortex, and thyroid.

Tissue sections from Ctrl, EAT, and EAT+GL mice were stained for CD4 (green), IL-17A (red), and DAPI (blue). Hippocampus (A); Cortex (B); Thyroid (C). Left panels show low-magnification representative images with yellow boxes indicating regions of interest; middle panels show corresponding high-magnification views. Right panels display single-channel images for DAPI, CD4, and IL-17A, highlighting the spatial overlap of $CD4^{+}$ cells with IL-17A signal (appearing yellow/orange in merged images).

Ctrl

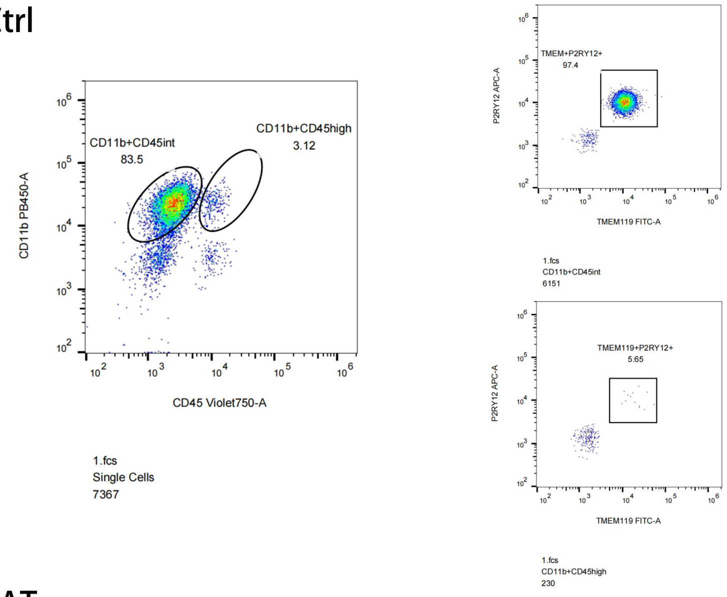

EAT

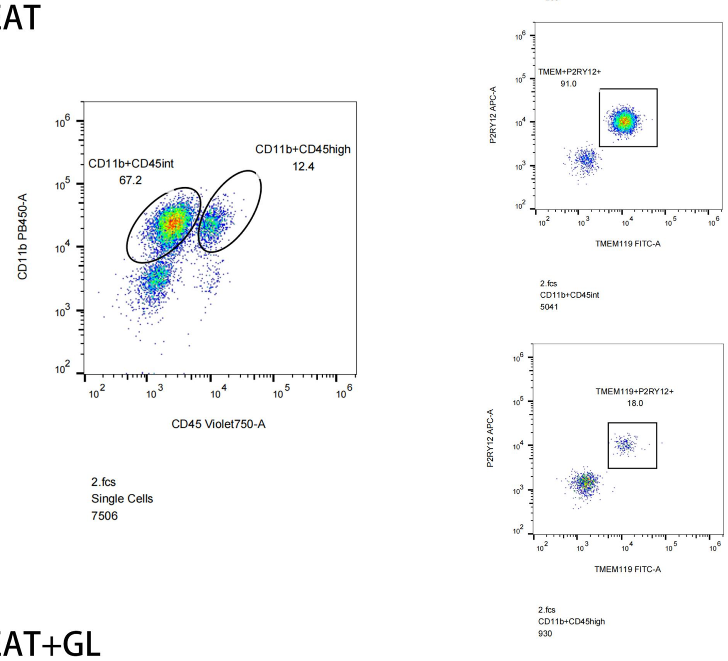

EAT+GL

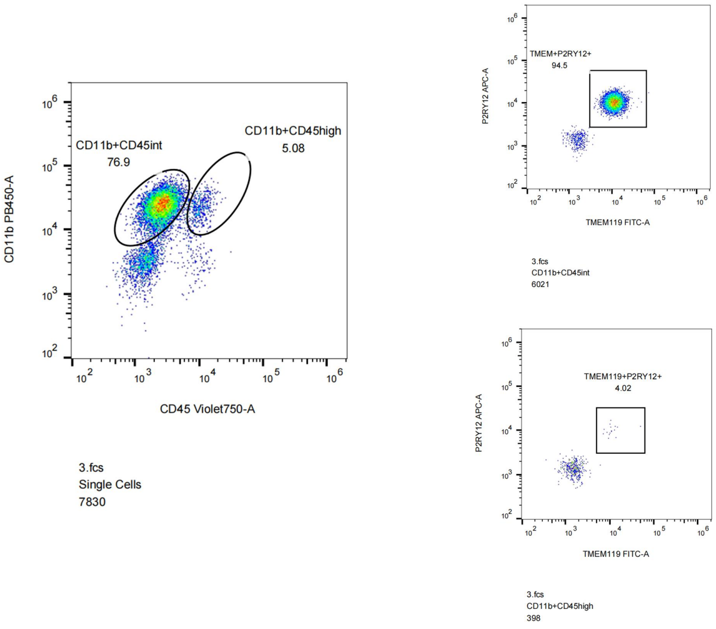

Figure. S7 Representative flow cytometry gating strategy for myeloid subsets and TMEM119/P2RY12 expression in Ctrl, EAT, and EAT+GL groups.

Single cells were gated first, followed by identification of $CD11b^{+}$CD45^int and $CD11b^{+}$ CD45^high populations on CD11b vs CD45 plots. Within each population, TMEM119 (FITC) and P2RY12 (APC) were used to gate $TMEM119^{+}P2RY12^{+}$ cells.

Numbers indicate the percentage of events within each gate.
